# Massive open online courses in health sciences from Latin American institutions: A need for improvement?

**DOI:** 10.12688/f1000research.11626.1

**Published:** 2017-06-19

**Authors:** Carlos Culquichicón, Luis M. Helguero-Santin, L. Max Labán-Seminario, Jaime A. Cardona-Ospina, Omar A. Aboshady, Ricardo Correa

**Affiliations:** 1 Emerge, Emerging Diseases and Climate Change Research Unit, School of Public Health and Administration, Universidad Peruana Cayetano Heredia, Lima, 15102, Peru; 2 Facultad de Ciencias de la Salud, Universidad Nacional de Piura, Piura, 20001, Peru; 3 Public Health and Infection Research Group, School of Medicine, Faculty of Health Sciences, Universidad Tecnológica de Pereira, Pereira, 660003, Colombia; 4 Infection and Immunity Research Group, School of Medicine, Faculty of Health Sciences, Universidad Tecnológica de Pereira, Pereira, 660003, Colombia; 5 Clinical Pharmacology Department, Faculty of Medicine, Menoufia University, Menoufia, 32721, Egypt; 6 Warren Alpert School of Medicine, Brown University, Providence, RI, 02914, USA

**Keywords:** Health education, Education distance, Continuing education, Latin America

## Abstract

**Background:** Massive open online courses (MOOCs) have undergone exponential growth over the past few years, offering free and worldwide access to high-quality education. We identified the characteristics of MOOCs in the health sciences offered by Latin American institutions (LAIs).

**Methods:** We screened the eight leading MOOCs platforms to gather their list of offerings. The MOOCs were classified by region and subject. Then, we obtained the following information: Scopus H-index for each institution and course instructor, QS World University Ranking® 2015/16 of LAI, and official language of the course.

**Results:** Our search identified 4170 MOOCs worldwide. From them, 205 MOOCs were offered by LAIs, and six MOOCs were health sciences related. Most of these courses (n = 115) were offered through Coursera. One health science MOOC was taught by three instructors, of which only one was registered in Scopus (H-index = 0). The remaining five health science MOOCs had solely one instructor (H-index = 4 [0–17]). The Latin American country with the highest participation was Brazil (n = 11).

**Conclusion: **The contribution of LAI to MOOCs in the health sciences is low.

## Introduction

The 21 ^st^ century technological and educational revolution has increased access to massive open online courses (MOOCs). They are internationally available online educational courses that are delivered using Web 2.0. MOOCs incorporate video conferencing supports and allow individuals worldwide to access high quality content provided by top-ranking universities ^1^. A large number of users have participated in more than 3859 MOOCs through the most popular platforms, such as Coursera®, edX® and Udacity® ^2^.

Furthermore, MOOCs have generated interest because of their innovative educational techniques ^3^. The international recognition of the quality of education, the flexible schedules and the absence of geographical barriers motivates students to access MOOCs ^4, 5^. In fact, they represent one strategy to reduce costs and enable continuous medical education, especially to rural physicians of developing countries ^6, 7^.

Despite their proven pedagogical quality and their impact, participation in MOOCs is lower from Latin American countries compared to USA or Europe because of difficulties accessing the technology, language barriers and low offering from Latin American institutions (LAIs) ^3, 8^. This study aimed to identify the characteristics of MOOCs offered by LAIs in the health science field.

## Methods

A search of MOOCs was performed using the virtual institutions catalog of eight platforms; Coursera®, edX®, FutureLearn®, Canvas.net®, MiriadaX®, iversity®, Open Education by Blackboard®, and NovoEd® from June 24 to June 30, 2016. These are the largest platforms and host more than 75% of MOOCs available worldwide ^9^. A search was conducted to identify MOOCs (cMOOCs and xMOOCs) that had current free access. Among these, we identified MOOCs that were offered by a LAI, and then identified which are related to health sciences. Each MOOC was screened for the location of educational institution (Latin America, non-Latin America), H-index of institution and instructor (provided by Scopus), QS World University Ranking® (QS) 2015/16 of the educational institution, official language of the course and subject of course (health sciences, non-health sciences). Categorical variables were summarized using frequencies and percentages, and numeric variables were summarized using median and range.

## Results

The search identified 4170 MOOCs offered by educational institutions worldwide. LAI offered 205 MOOCs (4.91%). Table 1 summarizes the results of each platform.

**Table 1.** Characteristics of massive open Online courses (MOOCs) offered by Latin American institutions (LAI).

Platform	Number (%) of MOOCs offered by LAI	Number (%) of LAI offering MOOCs	Number (%) of health science MOOCs offered by LAI	Language of MOOCs offered by LAI, n (%)
**Coursera**	115 (56.1)	10 (21.28)	1 (16.67)	Spanish: 86 (74.78)
				Portuguese: 29(25.22)
**Canvas.net**	0 (0)	0 (0)	0 (0)
**edX**	21 (10.24)	3 (6.38)	3 (50)	Spanish: 21 (100)
**MiriadaX**	46 (22.44)	28 (59.57)	2 (33.33)	Spanish: 38 (71.42)
				Portuguese: 8 (28.57)
**FutureLearn**	3 (1.46)	1 (2.13)	0 (0)	Spanish: 1 (33.33)
				English: 2(66.67)
**NovoEd**	0 (0)	0 (0)	0 (0)
**iversity**	0 (0)	0 (0)	0 (0)	
**Open** **Education by** **Blackboard**	20 (9.76)	5 (10.64)	0 (0)	Spanish: 12 (60)
				Portuguese: 8 (40)

Only six (2.93%) of these courses were in health sciences; one by Coursera®, two by Miriada X®, and three by edX®. One of the health science MOOCs was taught by three instructors, only one of whom was registered on Scopus and had an H-index of zero. The other five health science MOOCs offered by a LAI had only one instructor, and the median H-index was four (range, 0–17).

According to the number of institutions per country, Brazil contributed with 24.44% (n = 11), Colombia 22.22% (n = 10), Argentina 13.33% (n = 6), Mexico 13.33% (n = 6), and Peru 8.89% (n = 4), to the platforms studied.

The top-five LAIs with the most MOOCs in the platforms were Monterrey Institute of Technology and Higher Education, Mexico 17.83% (n = 33, H-index = 71, QS = 238°), National Autonomous University of Mexico, Mexico 16.21% (n = 30, H-index = 68, QS = 160°), Universidad de los Andes, Colombia 9.18% (n = 17, H-index = 92, QS = 283°), Ministry of Health Mexico, Mexico 6.48% (n = 12, H-index, QS = not available), Technological Institute of Aeronautics, Brazil 5.94% (n = 11, H-index = 56, QS = not available).

Massive open online courses in health sciences from Latin American institutions in 2016 Click here for additional data file. Copyright: © 2017 Culquichicón C et al. 2017 Data associated with the article are available under the terms of the Creative Commons Zero "No rights reserved" data waiver (CC0 1.0 Public domain dedication).

## Discussion

The number of MOOCs offered by LAIs was low compared with other regions. They represented almost the 5% of the MOOCs offered by educational institutions worldwide, in contrast with US institutions that offers most of these courses among several platforms ^1^.

Brazil and Mexico offer the most available MOOCs from Latin America. This could be due to the higher demand for MOOCs in these countries, especially in Brazil, which is related with a broad multidisciplinary research culture that can foster a high user demand among undergraduates ^10^.

Additionally, there was a low number of health sciences MOOCs offered by LAIs. Mexico offered the largest number of MOOCs in health sciences, which may be attributed to the cutting-edge educational strategies and individuals with high academic degrees available ^11^. It is worth mentioning that some organizations, like World Medical Association and the Internet Medical Society, are establishing agreements with some LAIs to develop high quality MOOCs for the benefit of the medical community that works in rural areas.

Even in developed countries, educational institutions that offer MOOCs want to achieve academic and scientific excellence. The currently offered MOOCs by LAIs are provided by instructors who have low H-indices, which may indirectly influence the quality of MOOCs ^12^. This may be due to a low level of training of the faculties and deans of health sciences schools in LAIs, and lack of incentives for undertaking teaching and research activities in these institutions ^13^.

This study has some limitations, such as the lack of data concerning instructors in some platforms, and the incomplete coverage of all available platforms. However, the covered platforms represent only 75% of worldwide MOOC, to the best of our knowledge, this is the first study with greater coverage in the scientific community ^5^. Despite the limitations that the H-index has, it´s the only indirect quality measure available for notifying the expertise of the instructors ^14^.

## Conclusion

The contribution of LAIs to health science MOOCs is low. LAIs should invest, develop, and promote this type of educational strategy, which offers huge potential for continuing medical education in this century, and promote access to these technologies, particularly in rural and remote areas.

## Data availability

The data referenced by this article are under copyright with the following copyright statement: Copyright: © 2017 Culquichicón C et al.

Data associated with the article are available under the terms of the Creative Commons Zero "No rights reserved" data waiver (CC0 1.0 Public domain dedication). 

Dataset 1: Massive open online courses in health sciences from Latin American institutions in 2016. doi, 10.5256/f1000research.11626.d164891 ^15^
