# Supplementary material for: Massive open online courses in health sciences from Latin American institutions: A need for improvement?
Source: F1000Res. 2017 Jun 19;6:940. [Version 1] doi: 10.12688/f1000research.11626.1 (PMC5499794; doi:10.12688/f1000research.11626.1)
Supplement: Peer review history

# Peer review: Referee response for version 1

**DOI:** 10.5256/f1000research.12557.r23579

**Published:** 2017-06-30

**Authors:** Ritu Madan

**Affiliations:** 1 Department of Internal Medicine, Froedtert Hospital, Medical College of Wisconsin, Milwaukee, WI, USA

It is a good research idea and a thoroughly done research but that being said, I am not sure if this is relevant to the scope of this website. It will be interesting to see how the quality of MOOC in Latin America compare with the rest of the world though.

I have read this submission. I believe that I have an appropriate level of expertise to confirm that it is of an acceptable scientific standard.

# Peer review: Referee response for version 1

**DOI:** 10.5256/f1000research.12557.r23578

**Published:** 2017-06-29

**Authors:** Sri Harsha Tella

**Affiliations:** 1 National Institute of Child Health and Human Development (NICHD), National Institutes of Health (NIH), Bethesda, MD, USA; 2 University of South Carolina, Columbia, SC, USA

This is an unique and excellent study that is focused on open online courses in Latin American medical education.

 Minor comments: It would be great if authors can comment on:

- Why Latin America is having low rate of medical online courses and potential answers for these problems? - For example: Many universities in USA have free provision of internet access to medical students while in campus and students are made aware of the available courses in many possible ways. Do they have this kind of feature in Latin American countries? If not- it may be one of the contributing factors. This opens doors to many questions- like the fee charged in USA medical schools vs the fee charged by Latin American medical schools -- one of the few potential factors that may be contributing to the difference. 

I have read this submission. I believe that I have an appropriate level of expertise to confirm that it is of an acceptable scientific standard.
